# Prediction of Stature From Percutaneous Anthropometric Dimensions of the Femur and Tibia Among Adult Nigerians

**DOI:** 10.7759/cureus.96603

**Published:** 2025-11-11

**Authors:** Ahmed A Salawu, James O Ikpa, Maryam O Raji-Salawu

**Affiliations:** 1 Department of Anatomy, College of Medicine, University of Lagos, Lagos, NGA; 2 Department of General and Colorectal Surgery, Lagos University Teaching Hospital, Lagos, NGA; 3 Department of Family Medicine, Palliative Care Unit, Lagos University Teaching Hospital, Lagos, NGA

**Keywords:** anthropometry, femur, regression models, stature, tibia

## Abstract

Introduction: Estimating stature, sex, ancestry, and age is central to forensic anthropological profiling, underpinning human identification in medico-legal contexts and mass-casualty events. Skeletal analysis informs these components. Although widely established in developed settings, Nigeria and many developing countries lack robust forensic anthropometric datasets despite rising disaster rates. This study sought to derive population‑specific regression equations to estimate stature from percutaneous femoral and tibial measurements in Nigerians. Regression analysis has proven to be the most straightforward and dependable approach for estimating stature.

Methods: This cross‑sectional observational study was conducted among 255 healthy Nigerian adults (130 males, 125 females; 18-65 years) recruited by stratified random sampling from the University of Lagos and Lagos University Teaching Hospital, Lagos, Nigeria, following ethical approval (approval number: CMUL/HREC/0955/19). Stature, femoral length, tibial length, and femoral bi‑epicondylar width were measured using standardized International Society for the Advancement of Kinanthropometry (ISAK) protocols with calibrated instruments (SECA™ stadiometer (Hamburg, Germany), Rosscraft calipers (Campbell, Canada), and Mitutoyo vernier calipers (Kawasaki, Japan). All measurements were taken by a single investigator at fixed times to minimize bias; intra‑observer reliability was assessed by triplicate readings, with mean values recorded. Bilateral measurements were averaged, and outliers were excluded if attributable to error or implausibility. Data were analyzed in IBM SPSS Statistics software, version 25 (IBM Corp., Armonk, NY) after normality and regression assumptions were verified, and sex‑specific and pooled regression models were developed to predict stature.

Results: The mean height of males was higher than females, reflecting clear sexual dimorphism in stature. Regression analysis demonstrated strong, statistically significant correlations between stature and femoral/tibial dimensions in both sexes. The pooled models yielded high coefficients of determination (R²) with low standard errors of estimate, indicating good predictive accuracy, with tibial length emerging as the most reliable predictor of stature, offering the greatest accuracy across both sexes (males standard error of estimate (SEE) ± 5.08 cm; females SEE ± 16.02 cm), whereas femoral intercondylar width contributed little to predictive value.

Conclusion: This study establishes a significant correlation between lower limb dimensions and stature, aligning with trends reported in other groups. The dataset generated offers a valuable forensic reference to aid human identification, particularly in contexts involving fragmented or mutilated remains. By providing population‑specific standards, this work enhances the application of lower‑limb metrics in forensic practice within Nigeria. There is a need to broaden research across diverse Nigerian ethnic groups, conduct cross-validation on skeletal remains, and integrate advanced analytical tools to enhance applicability. Also, incorporating artificial intelligence and computational modelling offers further potential to refine accuracy and strengthen cross‑validation of regression models.

## Introduction

Identification of the deceased is a fundamental aspect of post‑mortem examinations and is often required in cases of suicide, natural disasters, and mass‑casualty incidents [1,2]. Forensic anthropologists play a central role in this process by reconstructing the biological profile of individuals from skeletal evidence, with sex and stature estimation being primary objectives.

Stature, a key biological characteristic, is particularly valuable in personal identification. Its estimation from skeletal remains has long been recognized as an essential tool in medico-legal investigations and mass disaster scenarios [3]. When direct measurement of height is not feasible, such as in debilitated individuals, those confined to bed rest, or cases involving spinal or limb pathology, long bones like the femur and tibia provide reliable alternatives for stature reconstruction [4].

Historically, a wide range of skeletal elements, from the femur to smaller bones such as metacarpals, have been employed for stature estimation. While smaller elements can yield reasonably accurate results, long bones consistently demonstrate superior predictive value [2,5]. Even bone fragments, including proximal or distal ends, have been used, though complete long bones remain the preferred standard due to their higher accuracy.

In Nigeria, the need for reliable stature estimation methods is particularly pressing. The country has one of the highest road traffic fatality rates in Africa, with the World Health Organization reporting 33.7 deaths per 100,000 annually, accounting for one in four road accident deaths on the continent [6]. Such incidents, along with natural disasters, bombings, and politically motivated violence, often result in dismembered remains that complicate identification [7]. Despite this, forensic anthropological data specific to Nigerian populations remain scarce. While stature estimation methodologies are well established in developed countries [8], population‑specific datasets are limited in many developing nations [9].

In contexts where skeletal collections are unavailable, percutaneous anthropometric measurements from living individuals provide a practical alternative for developing regression models [10]. This approach allows for rapid, noninvasive stature estimation and is particularly valuable in regions such as Nigeria, where skeletal reference collections are lacking. Recent studies in Asia and Africa have demonstrated strong correlations between percutaneous long‑bone measurements and stature, with regression models showing predictive accuracies comparable to skeletal standards [3,11]. However, findings vary across populations due to differences in geography, ethnicity, and methodology, limiting their generalizability [12].

In Nigeria, only limited work has been conducted using percutaneous measurements, with most studies focusing on the upper limb [7]. Data on lower‑limb dimensions remain sparse, despite their recognized accuracy in stature prediction.

Accordingly, this study was designed to develop and validate regression models for stature estimation using percutaneous measurements of the femur and tibia in adult Nigerian males and females. By generating sex- and side-specific equations, the research aims to provide population-appropriate tools that address the current paucity of anthropometric data in Nigeria. Beyond their statistical value, these models are intended to strengthen forensic and medico-legal practice by offering reliable, scientifically defensible methods for biological profiling in contexts such as disaster victim identification, criminal investigations, and judicial proceedings. While percutaneous methods offer a valuable substitute for skeletal data, potential challenges, including inter‑observer variability, difficulty in locating landmarks in obese individuals, and measurement accuracy, must also be acknowledged.

## Materials and methods

Study design and ethical approval

This was a cross‑sectional observational study conducted among adult Nigerians aged 18-65 years, comprising students and staff of the College of Medicine, University of Lagos, and Lagos University Teaching Hospital, Lagos, Nigeria. Ethical clearance was obtained from the Research and Ethics Committee of the College of Medicine, University of Lagos (approval number: CMUL/HREC/0955/19).

Sample size and participant selection

Sample size was determined a priori using standard power calculations for regression analysis, ensuring adequate precision to detect moderate correlations between stature and long‑bone dimensions at 80% power and a 5% significance level. Participants were recruited through stratified random sampling to ensure representation across sex and major ethnic groups. Eligibility criteria included Nigerian origin, good general health, and absence of skeletal deformities, neurodegenerative conditions, or movement disorders. Individuals who declined to provide written informed consent were excluded.

Recruitment and consent

Recruitment was carried out through announcements in classrooms, hostels, and offices, supplemented by one‑on‑one invitations. All participants received detailed information about the study objectives, procedures, and potential benefits. Written informed consent was obtained, with assurances of confidentiality and the right to withdraw at any stage without consequence.

Measurement protocol and reliability

Anthropometric measurements (stature, femoral length, medial and lateral tibial length, and femoral bi‑epicondylar width) were obtained following standardized protocols established by the International Society for the Advancement of Kinanthropometry (ISAK) [13]. All measurements were performed in the anthropology laboratory of the Department of Anatomy at a fixed time of day to minimize diurnal variation. To reduce observer bias, a single trained investigator conducted all measurements.

The instruments used included a stadiometer (Alpha 220, SECA™, Hamburg, Germany), a large sliding caliper (Rosscraft™, Campbell, Canada), an anthropometry kit caliper 20 (Rosscraft™), spreading calipers, and vernier calipers (Mitutoyo™, Kawasaki, Japan). All devices were calibrated in centimeters to the nearest 0.1 cm, and calibration was verified prior to each measurement session.

Intra‑observer reliability was assessed by repeating each measurement three times under identical conditions, with the mean value recorded for analysis. For bilateral measurements, left and right values were averaged. Outliers were identified using standardized residuals (>±3) and excluded only when attributable to measurement error or biological implausibility.

Data handling and statistical analysis

Data were entered into Microsoft Excel 2019 (Microsoft Corporation, Redmond, WA) and analyzed using IBM SPSS Statistics software, version 25 (IBM Corp., Armonk, NY). Normality of distributions was assessed using the Shapiro-Wilk test, and regression assumptions (linearity, homoscedasticity, independence, and absence of multicollinearity) were verified prior to model construction. Simple and multiple linear regression analyses were performed to develop sex‑specific and pooled predictive equations for stature. Model performance was evaluated using the coefficient of determination (R²) and standard error of estimate (SEE).

Measurements

Stature

**Figure 1:**
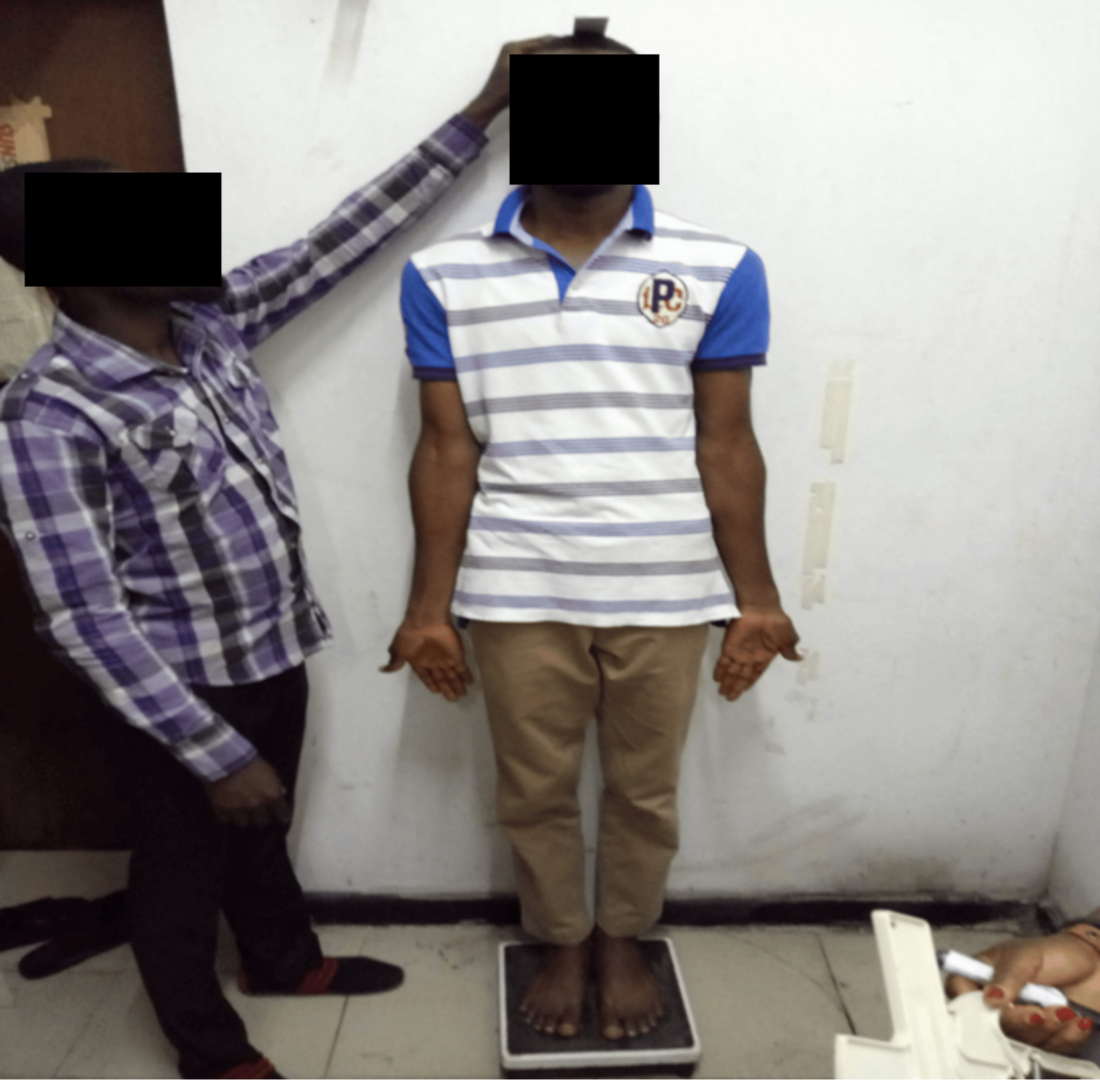
Measurement of stature

The measurement was taken as the maximum vertical distance from the floor to the vertex of the head. Technically, the vertex is defined as the highest point on the skull when the head is held in the Frankfort plane. This position is achieved when the line joining the orbitale to the tragion is horizontal or at right angles to the long axis of the body. In making the stature measurement, the measurer asked the barefoot subject to stand erect with heels together, both heels touching the base of the stadiometer, and arms hanging naturally by the sides. The heels, buttocks, upper part of the back, and usually, but not necessarily, the back of the head were in contact with the vertical wall. The subject was then instructed to “look straight ahead” and “take a deep breath.” The subject’s heels remained flat while the measurer then brought the headpiece firmly down, crushing the hair and making firm contact with the vertex, and made a pencil mark on the paper tape level with the underside of the headpiece. The measurement was made before the subject exhaled. The subject was asked to step away from the wall, the headpiece was removed, and the vertical distance from the floor to the pencil mark on the stadiometer was measured (Figure 1).

Femur Length

This was the distance from the marked most palpable part of the greater trochanter to the marked most palpable part of the lateral epicondyle of the femur. The subject stood with feet together on the box. The right and left percutaneous femoral lengths were then measured.

Lateral Tibia Length

This is the distance from the marked most palpable part of the lateral condyle of the tibia to the marked most palpable part of the lateral malleolus. The subject stood with feet together on the box with both legs facing the anthropometrist. The right and left percutaneous lateral tibia lengths were then measured.

Medial Tibia Length

This is the distance from the marked most palpable part of the medial condyle of the tibia to the marked most palpable part of the medial malleolus. The subject sat on the box and crossed the right ankle over the left knee to present the medial surface of the right leg horizontally for measurement and vice versa for the left leg.

Femur Intercondylar Width

The distance between the medial and lateral epicondyles of the femur when the subject was seated and the leg was flexed at the knee to form a right angle with the thigh. The small bone caliper was applied, pointing downwards to bisect the right angle formed at the knee. The epicondyles were palpated by the third digit, starting proximal to the sites. The caliper pressure plates were applied firmly. If difficulty was encountered in locating the epicondyles, the third digit could search in a slightly circular motion, and caliper pressure plates could be manipulated slightly to ensure the sites are encompassed.

## Results

General considerations

To enhance measurement reliability, each parameter was recorded in triplicate under identical conditions, and the mean value was used for analysis. Statistical significance was set at p < 0.05. Model accuracy was evaluated using the SEE. All analyses were conducted with IBM SPSS Statistics version 23.0 (IBM Corp., Armonk, NY, USA). Descriptive statistics, including mean, range (minimum and maximum), standard deviation, and standard error, were computed separately for males, females, and the combined sample, as well as for left and right sides. These results are summarized in Table 1 (right side) and Table 2 (left side).

**Table 1:** Descriptive statistics for data used in sex and stature estimation for the right side Measurement unit: centimeters

		Mean±SD	Minimum	Maximum	Standard error
Right					
Femur length	Male	46.55±4.3	28.50	57.60	0.40
	Female	45.27±4.0	29.20	56.50	0.38
	Combined	45.94±4.2	28.50	57.60	0.28
Lateral tibia length	Male	42.91±2.8	29.80	48.70	0.26
	Female	41.74±2.7	35.20	47.20	0.26
	Combined	42.35±2.8	29.80	48.70	0.18
Medial tibia length	Male	41.26±2.9	26.40	48.60	0.27
	Female	40.05±3.1	28.20	46.60	0.30
	Combined	40.68±3.0	26.40	49.10	0.20
Intercondylar width	Male	8.03±1.0	5.50	10.00	0.09
	Female	8.20±3.1	5.10	38.90	0.30
	Combined	8.11±2.3	5.10	38.90	0.15

**Table 2:** Descriptive statistics for data used in sex and stature estimation for the left side Measurement unit: centimeters

		Mean±SD	Minimum	Maximum	Standard error
Left					
Femur length	Male	46.31±5.1	17	57.5	0.48
	Female	45.34±4.0	29	56.8	0.39
	Combined	45.80±4.6	17	57.5	0.31
Lateral tibia length	Male	42.91±2.6	37.2	48.9	0.24
	Female	41.73±2.8	35.2	47.7	0.27
	Combined	42.34±2.7	35.2	48.9	0.18
Medial tibia length	Male	41.17±2.9	27.6	47	0.26
	Female	40.01±3.1	28	46.3	0.3
	Combined	41.17±2.9	27.6	47	0.2
Intercondylar width	Male	8.00±1.0	5.8	10.2	0.09
	Female	8.16±3.2	5	39.7	0.31
	Combined	8.08±2.3	5	39.7	0.16

Bilateral asymmetry

To evaluate potential differences between contralateral measurements, paired‑sample t‑tests were conducted for each dimension (Table 3). This analysis was undertaken to determine whether statistically significant bilateral asymmetry existed, which would necessitate the development of side‑specific regression models for stature estimation.

**Table 3:** Paired t-test comparing left and right measured parameter. Measurement unit: centimeters

	Difference in mean (Right-Left)	Standard error (difference)	t-value	p-value	95%CI of difference
Femur length	0.142	0.143	0.994	0.321	-0.140, 0.424
Lateral tibia length	0.007	0.050	0.145	0.885	-0.091, 0.105
Medial tibia length	0.069	0.030	2.324	0.021*	0.010, 0.128
Intercondylar width	0.032	0.016	2.006	0.046*	0.001, 0.063

The results indicated that, in the majority of cases, mean differences between left and right sides were minimal, typically <1 mm and never exceeding 2 mm. The greatest degree of asymmetry was observed in medial tibial length (mean difference = 0.069 cm, t = 2.324, p < 0.05), followed by intercondylar width (mean difference = 0.032 cm, t = 2.006, p < 0.05). Both differences reached statistical significance, whereas all other measured dimensions did not differ significantly between sides.

Although the absolute magnitude of bilateral differences was small and unlikely to be of major biological relevance, side‑specific regression equations were nonetheless generated. This is because even subtle differences can carry forensic significance. In medico-legal identification, precision is critical, as stature estimates are often combined with other biological parameters to narrow the pool of possible matches. Small but statistically significant side‑specific variations, if overlooked, may introduce systematic error when applying regression models to fragmented or unilateral remains. By accounting for these asymmetries and providing side‑specific equations, this study enhances the reliability and defensibility of stature estimation in forensic casework.

This approach ensures methodological rigor and allows practitioners to apply the most accurate predictive model available. Accordingly, when applying the formulae derived in this study, it is essential to first identify the side of the body part where possible using appropriate anatomical landmarks and then apply the corresponding regression equation.

Sexual dimorphism

Independent two‑sample t‑tests were conducted to assess the presence of sexual dimorphism in the measured dimensions for both right and left sides (Tables 4, 5; right and left sides, respectively). Several variables demonstrated statistically significant differences between males and females (p < 0.05), including right femoral length, right medial and lateral tibial lengths, and left medial and lateral tibial lengths. In each case, males exhibited significantly greater mean values than females, confirming the presence of sexual dimorphism within the studied population. The most pronounced difference was observed in left lateral tibial length (male mean = 42.91 cm; female mean = 41.73 cm; t = 3.301; SEE = 0.356).

**Table 4:** Result of the test for sexual dimorphism for measured parameters for the right side Measurement unit: centimeters

	Difference in mean (male-female)	Standard error (difference)	t-value	p-value	95% CI of difference
Femur length	1.281	0.553	2.317	0.021*	0.191, 2.370
Lateral tibia length	1.170	0.369	3.170	0.002*	0.443, 1.898
Medial tibia length	1.210	0.402	3.013	0.003*	0.419, 2.001
Intercondylar width	-0.167	0.306	-0.544	0.587	-0.771, 0.437

**Table 5:** Result of test for sexual dimorphism for measured parameters for the left side Measurement unit: centimeters

	Difference in mean (male-female)	Standard error (difference)	t-value	p-value	95% CI of difference
Femur length	1.077	0.620	1.735	0.084	-0.146, 2.300
Lateral tibia length	1.176	0.356	3.301	0.001*	0.474, 1.878
Medial tibia length	1.162	0.397	2.925	0.004*	0.379, 1.945
Intercondylar width	-0.166	0.315	-0.527	0.598	-0.788, 0.455

Stature estimation

Pearson’s correlation analysis was employed to evaluate the strength of association between stature and each of the measured long‑bone dimensions, assessed separately for the left and right sides (Table 6). All variables demonstrated positive correlations with stature, indicating that increases in bone length were consistently associated with increases in overall body height. Among the predictors, medial tibial length exhibited the strongest relationship with stature (right: r = 0.374; left: r = 0.372), whereas intercondylar width showed only a weak association (right: r = 0.040; left: r = 0.028).

**Table 6:** Correlation between stature and measured parameter in the right and left lower limb. Measurement unit: centimeters

	Right		Left	
	Correlation coefficient (r)	p-value	Correlation coefficient (r)	p-value
Femur length	0.266	<0.001*	0.248	<0.001*
Lateral tibia length	0.325	<0.001*	0.339	<0.001*
Medial tibia length	0.374	<0.001*	0.372	<0.001*
Intercondylar width	0.040	0.554	0.028	0.678

Regression models for stature estimation

Simple Linear Regression

Simple linear regression equations were developed for each measured dimension, stratified by sex (male, female) for the right (Table 7) and left (Table 8) sides. The predictive significance of each variable was evaluated, and model fit was assessed using the SEE, which reflects the residual variation around the regression line: Y = B + MX, where Y = the stature, M = the regression coefficient, X = measured dimensions, and B = the constant value of the equation.

**Table 7:** Simple linear regression model for Individual measurement in the right limb SEE: standard error of estimates; r: correlation coefficient; R: coefficient of determination Measurement unit: centimeters

	Equation	p-value	SEE	r	R^2^
	Femur length (F)				
Male	133.73+ (0.919) F	<0.001*	6.32	0.815	0.665
Female	147.09+(0.399)F	<0.324	16.38	0.737	0.543
Combined	130.51+(0.882)F	<0.001*	13.29	0.779	0.608
	Lateral tibia length (LT)				
Male	110.63+(1.535)LT	<0.001*	6.07	0.837	0.701
Female	129.018+(0.87)LT	0.142	16.29	0.765	0.586
Combined	103.58+(1.593)LT	<0.001*	13.04	0.802	0.644
	Medial tibia length (MT)				
Male	112.81+(1.543)MT	<0.001*	5.89	0.866	0.750
Female	116.97+(1.203)MT	<0.001*	16.03	0.814	0.663
Combined	102.29+(1.689)MT	<0.001*	12.79	0.860	0.740
	Intercondylar (I) length				
Male	167.158+(1.162)I	0.105	7.36	0.708	0.502
Female	163.11+(0.248) I	0.628	16.44	0.633	0.401
Combined	169.06+(0.241)I	0.554	13.78	0.674	0.455

**Table 8:** Simple linear regression model for Individual measurement in the leftlimb SEE: standard error of estimates; R: coefficient of determination Measurement unit: centimeters

	Equation	p-value	SEE	R	R^2^
	Femur length (F)				
Male	143.89+(0.70)F	<0.001*	6.508	0.794	0.632
Female	146.08+(0.42)F	0.288	16.370	0.752	0.566
Combined	137.39+(0.73)F	<0.001*	13.36	0.766	0.588
	Lateral tibia length (LT)				
Male	98.58+(1.82)LT	<0.001*	5.803	0.895	0.801
Female	128.69+(0.87)LT	<0.001*	16.28	0.775	0.601
Combined	98.23+(1.72)LT	<0.001*	12.98	0.827	0.684
	Medial tibia length (MT)				
Male	110.38+(1.61)MT	<0.001*	5.878	0.880	0.775
Female	117.463+(1.19)MT	<0.020	16.02	0.837	0.701
Combined	101.91+(1.70)MT	<0.001*	12.801	0.849	0.722
	Intercondylar (I) width				
Male	171.23+(0.66)I	<0.001*	7.411	0.679	0.461
Female	163.44+(0.21)I	0.677	16.445	0.604	0.366
Combined	169.69)(0.17)I	0.678	13.79	0.633	0.401

In the combined sample, right medial tibial length produced the most accurate predictions, with the lowest SEE (±12.79 cm) and the highest explained variance (R² = 74%). Among males, left lateral tibial length yielded the strongest model (SEE = ±5.08 cm; R² = 80%), while in females, left medial tibial length was the most reliable predictor (SEE = ±16.02 cm; R² = 70%). By contrast, intercondylar width consistently performed poorly across all groups, with the highest SEEs (±7.41-16.45 cm) and the lowest explanatory power (R² = 37-46%).

Multiple Regression Models

Multiple linear regression models were developed using combinations of the measured skeletal dimensions, stratified by sex (male, female) and for the combined sample, and calculated separately for the left and right sides (Table 9). Consistent with previous findings by Howley et al. [14], the inclusion of multiple predictors improved model performance, and forward stepwise regression was employed to identify the most informative variables. For females and the combined sample, right‑sided measurements produced the strongest models, explaining 77% and 70% of the variance in stature, respectively, values that exceeded those obtained from single‑dimension regressions. In males, left‑sided measurements yielded the most accurate predictions, with an adjusted R² of 87%, again surpassing the explanatory power of individual predictors.

**Table 9:** Multiple linear regression model using measured parameters. R: coefficient of determination; FL: femur length; LT: lateral tibia; MT: medial tibia; IW: intercondylar width Measurement unit: centimeters

	Equation	p-value	Adjusted R^2^
	Right		
Male	89.198+ (0.513)FL +(0.670)LT +(0.618)MT + (1.140)IW	<0.001*	0.854
Female	129.052+ (-0.286)FL +(-0.879)LT +(2.085)MT + (0.272)IW	0.158	0.774
Combined	94.882+ (0.198)FL +(0.305)LT +(1.300)MT + (0.153)IW	<0.001*	0.705
	Left		
Male	83.723+ (0.416)FL +(1.066)LT +(0.491)MT + (0.945)IW	<0.001*	0.871
Female	127.274+ (-0.658)FL +(-0.658)LT +(1.905)MT + (0.192)IW	0.180	0.733
Combined	93.931+(0.176)FL +(0.451)LT +(1.219)MT + (0.056)IW	<0.001*	0.701

**Figure 2:**
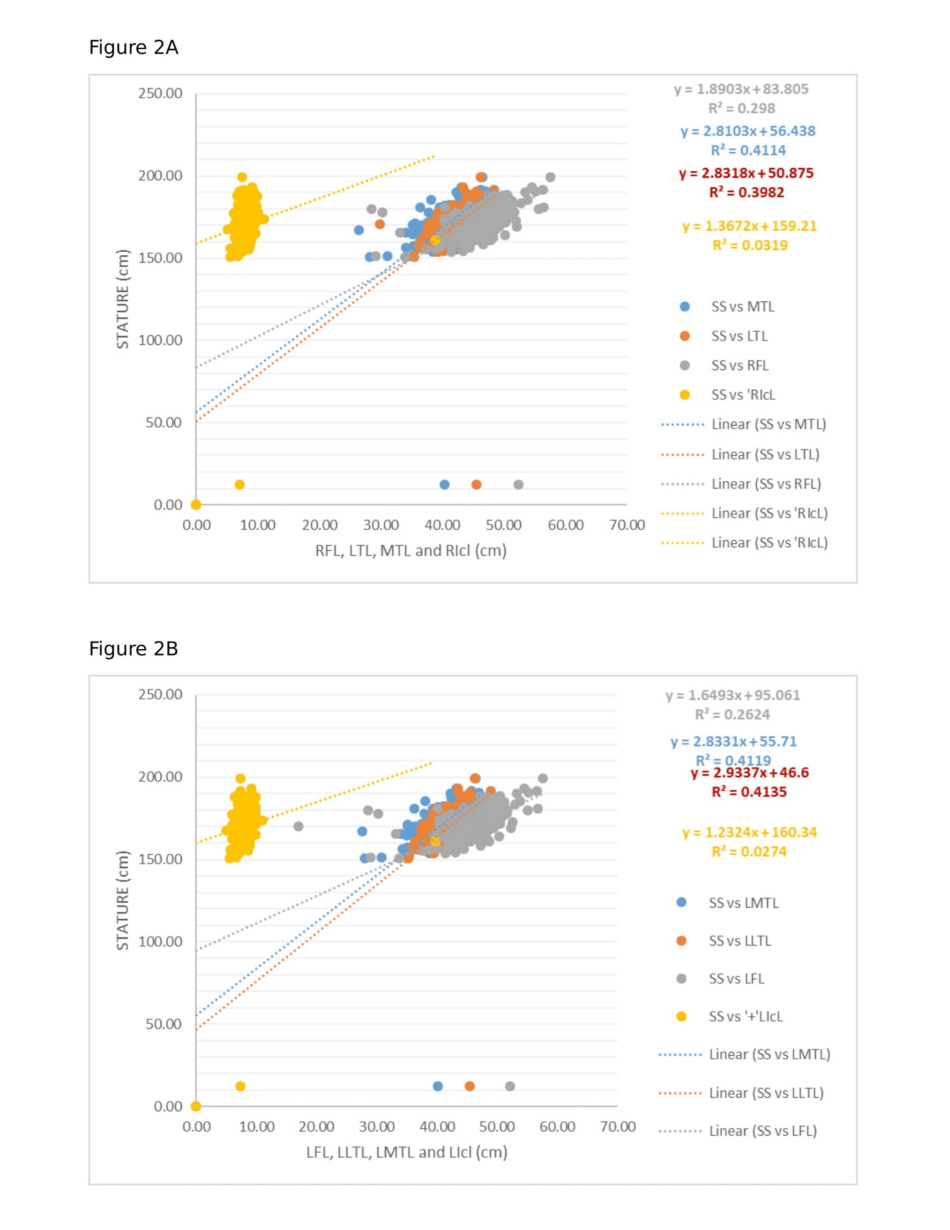
A: Scatter plot of stretched stature (SS) vs. right femur length (RFL), right lateral tibia length (LTL), right medial tibia length (MTL), and right intercondylar width (RICL). B: Scatter plot of stretched stature (SS) vs. left femur length (LFL), left lateral tibia length (LLTL), left medial tibia length (LMTL), and left intercondylar width (LICL).

Scatter plots (Figures 2A-2B) were generated to visually illustrate the strength of these associations.

## Discussion

Establishing the identity of the deceased is a fundamental component of postmortem examination [1], particularly in the aftermath of natural or human-induced disasters where reliable identification is often required [2]. Among the key biological parameters recoverable from skeletal remains, stature represents a critical trait for reconstructing the biological profile of an individual [15]. However, in contemporary Nigeria, there remains a notable scarcity of population‑specific data to support forensic anthropological research and practice, despite the rising incidence of mass‑fatality events [7]. The present study, therefore, sought to evaluate the potential of percutaneous anthropometric measurements of the femur and tibia as predictors of stature in adult Nigerian males and females. From a forensic and medico-legal perspective, the development of such regression models is of considerable importance: stature estimation not only contributes to narrowing the biological profile of unidentified individuals but also enhances the accuracy and defensibility of identifications in disaster victim recovery, criminal investigations, and judicial proceedings.

Sexual dimorphism in stature

The statistically significant difference in mean stature between males and females observed in this study reflects well-established biological mechanisms underlying sexual dimorphism. In females, the earlier onset of puberty and the associated surge in estrogen accelerate epiphyseal plate closure, thereby limiting longitudinal bone growth. In contrast, males typically experience a longer growth period and higher peak growth velocity, influenced by testosterone-driven increases in muscle mass and periosteal bone expansion. These hormonal differences are compounded by genetic factors regulating skeletal development, as well as environmental influences such as nutrition and physical activity during growth phases. For example, genes regulating growth and skeletal development may be differentially expressed between sexes, influencing height potential. Also, taller stature in males may have conferred advantages in mate selection, physical competition, or survival, reinforcing height differences over generations. Although the magnitude of dimorphism may vary across populations, the consistent pattern observed here aligns with findings from other African and non-African cohorts.

When compared with previous studies, the present findings agree with regional and international research [3,9,11,12,16]. Armah et al. [9] reported similar patterns in a Ghanaian population using percutaneous tibial and humeral lengths, while Nor et al. [16] demonstrated comparable results from osteological analyses of cadaveric bones. The convergence of evidence across both percutaneous and direct skeletal measurements underscores the robustness of long‑bone dimensions as indicators of stature and sex. Importantly, the tibia emerged as the most consistent and reliable predictor, a finding echoed in other African and non‑African populations.

From a forensic and medico‑legal perspective, these findings carry significant implications. In mass‑fatality incidents, criminal investigations, or cases involving dismembered remains, stature estimation forms a critical component of the biological profile. Even modest but statistically significant dimorphism can meaningfully narrow the pool of potential matches, particularly when combined with other anthropological markers such as age and sex. The regression models developed here provide population‑specific tools that address the current paucity of Nigerian anthropometric data, thereby enhancing the accuracy and defensibility of forensic identifications.

Correlation with stature

All lower‑limb dimensions examined in this study correlated positively with stature, with tibial measurements showing the strongest associations across sexes, while intercondylar width consistently displayed the weakest. These findings are consistent with previous research in both African [9,11] and non‑African populations [3,12,16], reinforcing the tibia’s role as a robust predictor of height regardless of whether percutaneous or osteological methods are used. Despite differences in methodology and geography, the predictive accuracies reported in these studies are broadly comparable to those observed here, underscoring the reliability of tibial length across populations.

From a forensic and medico-legal perspective, the tibia emerges as the most reliable contributor to regression models, offering precise stature estimates that can aid in biological profiling when remains are incomplete. Conversely, the limited predictive value of intercondylar width suggests it should not be used in isolation, though it may add marginal utility in multivariate models. By identifying the most informative skeletal parameters, this study strengthens the accuracy and defensibility of stature estimation in disaster victim identification, criminal investigations, and other medico-legal contexts.

Predictive value of lower‑limb dimensions

All lower‑limb dimensions examined in this study were positively associated with stature, with tibial length consistently emerging as the strongest predictor across sexes and the combined sample, while intercondylar width showed the weakest predictive value. These results are in line with previous studies in African and non‑African populations, where tibial length has repeatedly been identified as the most reliable indicator of height [10,11,16-18].

Ahmed [11], working with Sudanese Arab subjects, also reported significant positive correlations between stature and lower‑limb dimensions, with tibial length and foot length identified as the best predictors. The prediction accuracy in that study (SEE: 2.75-5.40 cm) was slightly lower but broadly comparable to the present findings. Similarly, Ozaslan et al. [10], in a Turkish cohort of medical students and staff, found tibial length to be the strongest predictor of stature, with SEE values ranging from 3.85 to 6.30 cm, again comparable to this study. Although some variation exists, such as the stronger role of the fibula and femur in Thai populations, as reported by Mahakkanukrauh et al. [19]. The minor variations observed across these studies likely reflect population‑specific genetic, environmental, and morphological differences, as well as methodological differences in measurement techniques. Such findings underscore the importance of developing regression models tailored to specific populations, since stature estimation is highly sensitive to regional and racial variation in skeletal proportions.

From a forensic and medico-legal perspective, these findings highlight the tibia, particularly its medial and lateral dimensions, as the most robust skeletal element for stature estimation in Nigerian adults. The relatively low SEE values in males suggest that tibial measurements can provide stature estimates with narrow error margins, strengthening their evidentiary value in human identification. Although the predictive accuracy was lower in females, the models still offer useful approximations when other skeletal elements are unavailable. Conversely, the weak performance of intercondylar width underscores its limited forensic utility as a standalone predictor.

Regression equations

Simple linear regression confirmed that individual lower‑limb dimensions could predict stature, but multivariate models incorporating multiple measurements produced higher coefficients of determination (R²) and lower error margins, particularly when stratified by sex. This demonstrates that sex‑specific multiple regression equations provide more accurate stature estimates than single‑dimension models, a finding consistent with earlier studies [11,16]. From a forensic and medico-legal perspective, the improved precision of multivariate models enhances the reliability of stature reconstruction in casework, including disaster victim identification and criminal investigations involving incomplete remains. The observed sex- and side-specific differences further highlight the need to tailor regression models to the biological context of the remains. In practice, the model with the lowest standard error of estimate should be applied to maximize accuracy, whether derived from simple or multiple regression, depending on the body parts available.

By establishing population‑specific regression equations, this study provides practitioners with scientifically defensible tools for estimating stature in medico‑legal contexts, including disaster victim identification, criminal investigations, and anthropological casework.

Model accuracy and forensic applicability

The regression models developed in this study demonstrated high predictive accuracy, with R² indicating strong correlations between stature and long‑bone dimensions, and SEE within the acceptable forensic tolerance. From a forensic perspective, this level of precision is sufficient to substantially narrow the pool of potential matches during human identification, particularly in mass‑casualty incidents or road traffic accidents where dismembered remains are common. Importantly, the use of population‑specific data addresses the limitations of applying non‑Nigerian formulae, which may introduce systematic bias due to inter‑population variation in body proportions. The applicability of these models extends beyond skeletal remains: because they are derived from percutaneous measurements, they can also be employed in medico-legal contexts involving living individuals where direct stature measurement is not feasible. Furthermore, the methodological rigor, including daily calibration of instruments, intra‑observer reliability checks, and verification of regression assumptions, enhances the defensibility of these models in legal proceedings, where methodological transparency is critical. While the absolute magnitude of bilateral asymmetry was small, the creation of side‑specific equations ensures that practitioners can apply the most accurate formula available, further strengthening the utility of these models in forensic anthropology.

Forensic implications

The regression models developed in this study provide a population‑specific framework for stature estimation in Nigerian adults, addressing a critical gap in forensic anthropology within the region. Their applicability is particularly relevant in disaster victim identification, road traffic accidents, and other medico-legal contexts where dismembered remains are frequently encountered. By offering reliable stature estimates from percutaneous long‑bone measurements, these models enhance the accuracy of biological profiling and reduce reliance on non‑representative formulae derived from other populations. Furthermore, the methodological rigor underlying their development strengthens their admissibility as expert evidence in legal proceedings, thereby contributing both to forensic practice and to the establishment of a Nigerian anthropometric reference database.

Limitations

Although the regression models developed in this study demonstrated strong predictive accuracy, several limitations should be acknowledged. First, the study was conducted in a single institution, and the sample was restricted to healthy Nigerian adults aged 18-65 years, which may limit generalizability to younger, older, or pathological populations. Second, while intra‑observer reliability was assessed, inter‑observer variability was not evaluated, and this may influence reproducibility in broader forensic practice. Third, percutaneous measurements can be challenging in obese individuals or in cases where anatomical landmarks are obscured, potentially reducing accuracy.

In addition, the regression equations were not externally validated or cross-validated, which constrains their broader applicability. Finally, the cross‑sectional design precludes assessment of temporal changes in body proportions.

As forensic anthropologists emphasize, regression models derived from one reference group may under- or overestimate outcomes when applied to other populations. Accordingly, the equations presented here are specific to this study population and are not intended for direct use in other regions. Nonetheless, they provide a useful reference point, and the methodology may be adapted for application in other populations.

Future directions

Beyond its immediate forensic applications, the study underscores the importance of expanding research to include additional Nigerian ethnic groups, validating methodologies on skeletal remains, and incorporating advanced statistical approaches, imaging technologies, and AI‑based models to enhance predictive accuracy and applicability. As Ogut [20] has emphasized, incorporating artificial intelligence and computational modelling could further refine predictive accuracy and enable robust cross‑validation of regression equations.

Future research should therefore expand sample size and diversity, incorporate inter‑observer reliability testing, and explore advanced statistical or machine‑learning approaches to refine predictive accuracy. Establishing a comprehensive, population‑specific anthropometric database for Nigeria would further strengthen the forensic applicability of stature estimation models in both medico‑legal and disaster victim identification contexts.

## Conclusions

This study investigated the relationship between stature and lower‑limb dimensions in an adult Nigerian population not previously been examined. Among the measured parameters, tibial length consistently showed the strongest correlation and predictive accuracy for stature, echoing findings from other populations. The resulting dataset provides a valuable forensic reference for identifying individuals, whether living or deceased, and will assist pathologists, anatomists, and medical examiners in cases involving dismembered or mutilated remains from both natural and man‑made disasters. Overall, this work strengthens the forensic utility of lower‑limb metrics by providing a reliable reference for human identification in the Nigerian context.
